# Sleep Disturbances Associated with Parkinson's Disease

**DOI:** 10.4061/2011/219056

**Published:** 2011-08-17

**Authors:** Keisuke Suzuki, Masayuki Miyamoto, Tomoyuki Miyamoto, Masaoki Iwanami, Koichi Hirata

**Affiliations:** Department of Neurology, Center of Sleep Medicine, Dokkyo Medical University School of Medicine, 880 Kitakobayashi, Mibu, Shimotsuga, Tochigi 321-0293, Japan

## Abstract

Sleep disturbances are common problems affecting the quality life of Parkinson's disease (PD) patients and are often underestimated. The causes of sleep disturbances are multifactorial and include nocturnal motor disturbances, nocturia, depressive symptoms, and medication use. Comorbidity of PD with sleep apnea syndrome, restless legs syndrome, rapid eye movement sleep behavior disorder, or circadian cycle disruption also results in impaired sleep. In addition, the involvement of serotoninergic, noradrenergic, and cholinergic neurons in the brainstem as a disease-related change contributes to impaired sleep structures. Excessive daytime sleepiness is not only secondary to nocturnal disturbances or dopaminergic medication but may also be due to independent mechanisms related to impairments in ascending arousal system and the orexin system. Notably, several recent lines of evidence suggest a strong link between rapid eye movement sleep behavior disorder and the risk of neurodegenerative diseases such as PD. In the present paper, we review the current literature concerning sleep disorders in PD.

## 1. Introduction

Parkinson's disease (PD) is a movement disorder characterized by bradykinesia, resting tremors, rigidity, and impaired postural reflexes, which are caused by the degeneration of dopaminergic neurons in the substantia nigra. However, the pathological course of PD has been recognized to be much more extensive, involving the serotoninergic, noradrenergic, and cholinergic systems [1]. These systems may play a role in the development of the nonmotor symptoms commonly observed in PD such as sleep disturbances, depression, olfactory dysfunction, cognitive impairments, fatigue, and autonomic dysfunctions. In a recent large study comprising 1,072 patients with PD, almost all of the patients exhibited at least one type of nonmotor symptoms [2].

Sleep disturbances are among the most common nonmotor symptoms, with a prevalence ranging from approximately 40% to 90%, and these disturbances can interfere with patients' quality of life [2–5]. Various factors, including nocturnal motor symptoms, psychiatric symptoms, dementia, dopaminergic medications, and circadian cycle disruptions, cause sleep disturbances [6]. Comorbidity with sleep apnea syndrome (SAS), restless legs syndrome (RLS), and rapid eye movement sleep behavior disorder (RBD) is often observed, complicating the sleep disturbances related to PD. The orexin system may be involved in PD, contributing to the daytime sleepiness independent of impaired sleep conditions. RBD preceding or coexisting with PD has received attention, but whether RBD and PD are caused by a similar neurodegenerative process remains unknown. The evaluation and treatment of sleep disorders in PD are of great importance because of their negative impact on quality of life. A “sleep benefit” of improved early-morning motor function before medication intake is often reported by some PD patients [7]. Högl et al. reported that levodopa concentrations and polysomnographic findings were similar between PD patients with and without the sleep benefit but that PD patients with the sleep benefit exhibited a different response profile to levodopa; the magnitude of motor deterioration after levodopa intake was greater in PD patients with the sleep benefit than in patients without it [8]. Subjective perceptions or sensory mechanisms may play a role in the sleep benefit in PD. In contrast, the effect of sleep deprivation on motor performance is controversial [9]. In this paper, we review and discuss the current literature concerning sleep disorders in PD.

## 2. Pathophysiology of Insomnia and Excessive Daytime Sleepiness

As a result of an examination of polysomnography (PSG) recordings, altered sleep structure has been observed in PD, namely, a decrease in the quantity of nonrapid eye movement (NREM) sleep stages 3 and 4 and REM sleep [10]. The degeneration of cholinergic neurons in the basal forebrain and brainstem including the pedunculopontine nucleus and noradrenergic neurons in the locus coeruleus results in disorders of REM sleep, and a loss of serotoninergic neurons in the raphe nucleus is associated with a reduction in the amount of slow-wave sleep [11]. In addition to the orexin and histamine systems, these serotoninergic, noradrenergic, and cholinergic neurons in brainstem serve as arousal systems that maintain wakefulness, and disturbance of these neurons leads to excessive daytime sleepiness. In patients with PD, a loss of orexinergic neurons in the posterior portion of the lateral hypothalamus [12] and a reduction in the number of A10 dopaminergic neurons in the ventral tegmental area [13] have been implicated in impaired wakefulness. The histaminergic neurons in the hypothalamus appear intact in patients with PD. Orexin/hypocretin may promote wakefulness by upregulating monoaminergic neuronal populations [14]. Wake-active dopaminergic neurons in the ventral periaqueductal gray matter have been identified [15] but seem to be intact in patients with PD [16]. In animal models, D2 receptors exhibit a biphasic response, with sedating effects occurring after low-dose stimulation of the presynaptic receptors and awakening effects occurring after high-dose stimulation of the postsynaptic receptors [17]. The ventral tegmental area and the mesolimbic and mesocortical dopaminergic circuits are crucial sites for the action of dopamine in the sleep-wake cycle [18]. In humans, low doses of dopaminergic stimulation may result in sleepiness, and high doses of stimulation may induce wakefulness, resulting in insomnia [19]. A placebo-controlled, randomized, double-blind, crossover study performed in 20 healthy volunteers using the multiple sleep latency test indicated that low-dose ropinirole reduces the time to sleep onset in humans [20]. By contrast, in a study of 54 consecutive levodopa-treated patients with PD referred for sleepiness, a positive correlation between mean daytime sleep latency and the daily administration of levodopa was observed, even after accounting for controlling factors, indicating that levodopa may have alerting effects in some groups of patients [21]. Nevertheless, all dopaminergic drugs can have sedating effects on patients with PD, and the mechanism of this discrepancy has not been fully elucidated [22].

## 3. Insomnia

**Figure 1:**
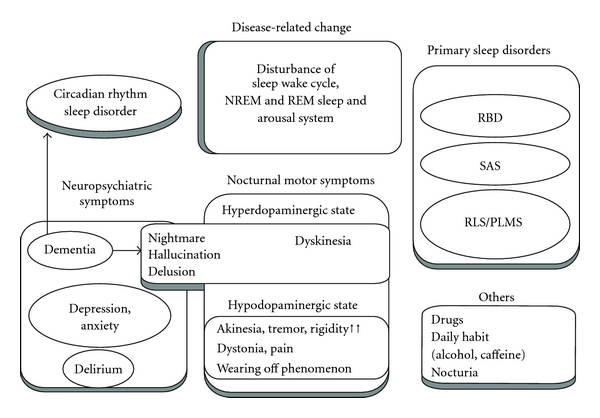
The major causes of insomnia and its contributing factors. RBD: rapid eye movement sleep behavior disorder; SAS: sleep apnea syndrome; RLS: restless legs syndrome; PLMS: periodic limb movement during sleep.

Insomnia is defined as a complaint of one or more of the following symptoms: difficulty falling asleep, difficulty staying asleep, early awakening, or nonrefreshing sleep that occurs despite adequate opportunities for sleep. Daytime impairments related to nighttime sleep difficulties have also been reported. In a community-based survey, sleep initiation was reported to be similar in all groups, but the PD patients complained of greater sleep fragmentation compared with diabetic patients or healthy control subjects [5]. In patients with PD, sleep maintenance insomnia (difficulty staying asleep) appears to be a common form of insomnia that is frequently caused by nocturnal motor disturbances [23, 24].  Figure 1 represents the major causes of insomnia observed in patients with PD. Insomnia is attributable to these various causes, but the prevalence of insomnia increases as the disease progresses along with the aging process, suggesting that disease severity has an impact on sleep disturbances [3, 23, 24]. Insomnia is probably correlated with depression, disease duration [24–26], or motor symptoms [3]. In contrast, the prevalence of insomnia was 54%–60% over an eight-year period in a prospective study, but the data showed no linear increase over an eight-year followup period [26]. This finding indicates that sleep disorders can occur during an early stage of PD.

Identifying the factors contributing to insomnia can result in an improvement in sleep. Melatonin administration to PD patients led to a significant improvement in subjective sleep disturbances, sleep quantity, and daytime sleepiness [27]. Using short-acting hypnotic drugs such as zolpidem [28], which have less impact on muscle relaxation, is recommended to prevent falls associated with sleep aids, especially in elderly subjects.

### 3.1. Nocturnal Disturbances

Lees and colleagues [4] have reported nocturnal disturbances in 215 of 220 PD patients, including nocturia (79%), difficulty turning over in bed (65%), painful muscle cramps (55%), nightmares (48%), limb or facial dystonia (34%), leg jerks (33%), and visual hallucinations (16%). Stack and Ashburn [29] studied impaired bed mobility in patients with PD and determined that approximately 80% of the patients turned in bed successfully but exhibited difficulty turning in bed, suggesting that identifying the least disruptive turning strategy may be useful. 

Chaudhuri et al. [23] developed the Parkinson's disease sleep scale (PDSS), a visual analogue scale that includes 15 PD-related nocturnal symptoms for assessing nocturnal disability in patients with PD. Their study showed that patients with an advanced level of the disease had impaired scores compared with those with early or moderate levels of the disease. The PDSS was described in a recent review [30] as a recommended, reliable scale, except that it may not be sufficient for screening for sleep apnea, RBD, or RLS. This scale has been validated and employed extensively in a number of countries and was reported to exhibit high reliability [24, 31–34]. Our multicenter study also found more severe nocturnal disturbances in patients with an advanced stage of PD, as measured by the PDSS, (Hoehn & Yahr (H&Y) stage IV) compared with those with early and moderate stages of the disease (H&Y I–III). These disturbances were associated with disease duration, depressive symptoms, and complications of dopaminergic treatments (such as dyskinesia and wearing-off symptoms) [24]. By contrast, Dhawan et al. [35] reported that nocturnal symptoms such as nocturia, nocturnal cramps, dystonia, and tremors were observed in untreated PD patients with short disease durations. PDSS-2, a new version of PDSS, has been developed, and its total and three domain scores, including disturbed sleep, motor symptoms at night, and PD symptoms at night, have been shown to correlate with patients' quality of life, the Unified Parkinson's Disease Rating Scale (UPDRS) motor scores, and disease severity in different patterns [36].

Nocturia is a common nighttime problem; 80% of PD patients exhibited two or more episodes of nocturia per night caused by overflow incontinence and a spastic bladder [4]. If the nocturia is found to be related to wearing-off symptoms, then changing medications to administer a long-acting dopamine agonist before bedtime can be beneficial. A urologic examination is recommended because nocturia may also be associated with the normal aging process or underlying urological diseases.

Nocturnal motor symptoms are caused by a hypodopaminergic state, such as akinesia and increased tremor and rigidity, and a hyperdopaminergic state, such as levodopa-related dyskinesia. The inability to turn in bed and difficulty in rising to pass urine during the night due to nocturnal akinesia are significant disabling symptoms. Increasing the dose of a dopamine agonist or levodopa or adding these drugs to the regimen of medications administered at bedtime should be considered. A double-blinded, placebo-controlled trial with 287 PD patients demonstrated the efficacy of 24-h rotigotine on daytime motor function (UPDRS part III) and nocturnal disabilities, as evaluated by the PDSS-2 [37]. In addition, subcutaneous overnight apomorphine infusion led to a dramatic reduction of nocturnal awakenings, nocturnal-off periods, pain, dystonia, and nocturia in patients with PD [38]. Arnulf et al. reported that high-frequency subthalamic nucleus stimulation in 10 PD patients with insomnia reduced nighttime akinesia by 60% and completely suppressed axial and early-morning dystonia [39]. 

By contrast, a reduction in the dose of dopaminergic drugs may be effective for the symptoms associated with a hyperdopaminergic state. If patients with frequent nocturnal awakenings have taken amantadine or selegiline, which have potential alerting effects, then a reduction in the dose of these drugs, discontinuation of the administration of these drugs, or a change in the time of administration of these drugs from evening to morning may reduce the number of nocturnal awakenings.

### 3.2. Assessment Tools for Sleep Disturbances in PD

PSG is the “gold standard” method used to evaluate sleep disorders and provides detailed information about actual sleep status, including sleep efficiency, sleep latency, and sleep structure. PSG can detect the cooccurrence of SAS, RBD, and periodic limb movements. However, the use of PSG is limited because of its cost and requirement for special equipment. As an alternative, questionnaire-based sleep studies have been widely conducted. The application of several scales for sleep disturbances has recently been reviewed [30]. The Pittsburgh Sleep Quality Index [40] is recommended to assess overall sleep abnormalities, and the Epworth sleepiness scale (ESS) is suggested for use in evaluating daytime sleepiness [41]. However, prior studies have reported that ESS score was correlated with multiple sleep latencies, but the correlation was weak and false negatives were detected, suggesting that a normal ESS score does not exclude the sleepiness observed in PD [21]. The Parkinson's disease sleep scale (PDSS) [23], a visual analogue scale including 15 PD-related nocturnal symptoms for assessing nocturnal disability in PD, is now a recommended, reliable scale [30]. The scale includes the following items: overall quality of nighttime sleep (item 1), sleep onset and maintenance insomnia (items 2 and 3), nocturnal restlessness (items 4 and 5), nocturnal psychosis (items 6 and 7), nocturia (items 8 and 9), nocturnal motor symptoms (items 10-13), sleep refreshment (item 14), and daytime dozing (item 15). For further improvement, the PDSS-2, a modified version of the PDSS that assesses the frequency of nocturnal symptoms and includes the screening of SAS, has been published with an excellent level of validity and reliability [36].

## 4. Excessive Daytime Sleepiness

After a report in 1999 stated that sudden-onset sleep episode is associated with motor vehicle accidents in PD patients who take nonergot dopamine agonists (either ropinirole or pramipexole) [42], the association of excessive daytime sleepiness (EDS) or sleep episodes with dopaminergic medications has become a focus of attention. Approximately 15%–50% of PD patients have been reported to show EDS [43–45]. A high Epworth sleepiness scale (ESS) score, male gender status, longer disease duration, and high-disease severity have also been observed to be associated with EDS [43, 44, 46]. Similar to the insomnia observed in patients with PD, multiple factors are associated with EDS: impaired arousal systems in addition to the disease process, dopaminergic medication, nocturnal disturbances, and concurrent primary sleep disorders such as SAS, RBD, and RLS are thought to be contributing factors. In addition, narcolepsy-like symptoms have been observed in patients with PD. Daytime sleepiness or sleep episodes exhibiting a short sleep latency, short sleep onset REM period, and decreased orexin levels are independent of the patients' nighttime sleep conditions. These symptoms are similar to those observed in narcolepsy, which is a sleep disorder characterized by severe daytime sleepiness and caused by loss of orexin neurons. However, cataplexy is lacking in patients with PD [47], and the role of orexin levels in PD patients with EDS is still controversial. Studies in PD patients with EDS have not observed a reduction in orexin concentrations in the cerebrospinal fluid [48, 49]. However, while markedly decreased orexin levels in the hypothalamus and a loss of orexin neurons have been observed in PD patients and were significantly correlated with clinical disease progression, no description was provided for EDS [12, 50]. The lumbar cerebrospinal fluid may not reflect the orexin cell loss reported in the hypothalamus of patients with PD. Further research is needed to determine whether a decrease in the number of orexin neurons in the hypothalamus or other systems, in addition to orexin dysfunction, accounts for EDS in PD patients. Additional work is also required to determine whether decreased orexin levels reflect disease-related changes or secondary, compensatory changes that result from dopaminergic dysfunction [51, 52]. 

Several studies have demonstrated that taking dopamine agonists or levodopa is associated with increased daytime sleepiness in patients with PD [43, 44, 46, 53, 54]; however, several other studies have failed to confirm this significant association [55, 56]. To date, whether specific dopamine agonists are associated with sleepiness is still unclear [43, 44, 54]. Sudden onset sleep episodes while driving have been reported in 3.8%–22.8% of PD patients and are associated with a high score on the ESS [43, 46, 56]. This finding suggests that PD patients with high ESS scores are at risk for experiencing sleep episodes while driving.

## 5. Comorbid Sleep Disorders

### 5.1. Rapid Eye Movement Sleep Behavior Disorder

Rapid eye movement sleep behavior disorder (RBD) is characterized by a loss of muscle atonia during REM sleep that results in dream-enacting behavior, which often leads to injury to the individual or bed partner [57]. RBD tends to affect older individuals and has a higher prevalence in males [58]. 

Lesions of the locus coeruleus perialpha in cats and the sublaterodorsal nucleus in rats have been suggested to cause REM sleep without atonia and with complex movements [59, 60]. Involvement of subcoeruleus-coeruleus complex was found in cases with incidental Lewy body disease (preclinical stages of PD) [61]. This brain region may be crucial for RBD pathophysiology in addition to the cholinergic nuclei, pedunculopontine nucleus, and laterodorsal tegmental nucleus, which play a role in regulating REM sleep. A flip-flop switch model for the control of REM sleep has been proposed: GABAergic REM-on neurons located in the sublaterodorsal nucleus inhibit GABAergic REM-off neurons located in the ventrolateral periaqueductal gray matter and lateral pontine tegmentum, and vice versa [62]. 

RBD was considered to be an idiopathic, isolated disorder until a study in 1996 by Schenck demonstrated that 38% of 29 patients with idiopathic RBD developed PD after a mean followup of 3.7 ± 1.4 years [63]. Subsequently, a positive association has emerged between RBD and neurodegenerative disorders, particularly synucleinopathies such as PD, multiple system atrophy, and dementia with Lewy bodies [58, 64, 65]. Early manifestations of PD preceding the onset of typical motor symptoms, such as impaired visual and olfactory discrimination, cardiac sympathetic denervation, and cognitive impairment, have been observed in idiopathic RBD patients [66–70]. As a possible prodromal phase of neurodegenerative diseases, a diagnosis of RBD is crucial for early intervention to treat neurodegenerative disorders. Therefore, to establish an accurate diagnosis, a quantitative visual scoring of the electromyographic (EMG) data in REM sleep may be necessary [71]. In a recent report, RBD preceded the onset of synucleinopathies by up to 50 years [72], indicating that if idiopathic RBD patients lived long enough, the underlying cause of the neurodegenerative disease would be unveiled [73]. 

Recently, Iranzo et al. [74] conducted a study measuring PSG at baseline and after a mean followup of five years; this study revealed that excessive tonic and phasic EMG activity occurs during REM sleep and is increased over time in patients with RBD. This finding suggests that an underlying progressive process affects the brainstem in patients with RBD. Even though as many as half of RBD patients will develop neurodegenerative diseases, a wide variability is observed in the incidence rates of PD development, and no method exists to predict which patients will develop PD. We cannot currently predict why some idiopathic RBD patients develop PD and others do not. However, a recent study by Postuma et al. has helped elucidate this subject. Their results indicate that in subjects with idiopathic RBD initially free of neurodegenerative disease, the severity of the REM atonia loss on the baseline PSG findings can predict PD development [75].

In terms of the comorbidity of PD and RBD, PD patients with RBD exhibited a predominantly nontremor phenotype, an increased frequency of falls, and a poor response to dopaminergic medications, which were associated with orthostatic hypotension and impaired color vision. However, overall disease severity, quantitative motor testing, and motor complications did not differ between PD patients with RBD and those without RBD [76, 77]. Interestingly, restored motor control (movements, speech, and facial expressions) has been observed during REM sleep with enacted dreams in PD patients who had RBD [78]. 

RBD can be triggered by antidepressants, such as tricyclics, selective serotonin reuptake inhibitors, and serotonin-norepinephrine reuptake inhibitors; by beta-blockers; by states of barbiturate and alcohol withdrawal [79]; however, whether the subjects who develop RBD are susceptible to drugs or whether they have underlying diseases has not been fully elucidated. Clonazepam (0.5 to 1.5 mg) at bedtime is effective for decreasing the frequency and severity of RBD; however, it has little effect on EMG tone [80]. Melatonin has been indicated to ameliorate RBD symptoms [81] and improve EMG tone during REM sleep [82]. Levodopa and pramipexole also reduce the clinical manifestations of RBD [80], although in a prospective study of 11 consecutive PD patients with RBD, pramipexole improved parkinsonism but did not modify RBD [83]. The administration of the herbal medication Yi-Gan San at 2.5 g three times a day, alone or in conjunction with 0.25 mg clonazepam, has been reported to be effective in treating RBD [84]. However, there have been no randomized, double-blind, placebo-controlled trials on the treatment for RBD in the PD population.

RBD may be a prodrome of neurodegenerative disorders, including PD, and this notion is a topic of great interest. As a result, the following questions are under investigation: what factors can determine who will develop neurodegenerative disorders? who will remain free of symptoms during life? what neuroprotective strategy is effective for patients who are susceptible to neurodegenerative disorders? and when should this strategy be employed?

### 5.2. Restless Legs Syndrome

Restless legs syndrome (RLS) and PD are considered to share pathophysiological characteristics, given that both neurological disorders exhibit favorable responses to dopaminergic medications; however, RLS usually responds to lower doses than those required for PD. In addition, several studies have demonstrated a higher rate of comorbidity of RLS and PD compared with the prevalence of idiopathic RLS in the general population [3, 85], while other studies have reported no difference in the prevalence of RLS when comparing PD patients with the general population [86, 87]. However, one should note that daytime dopaminergic medications for PD may unmask subclinical RLS by augmentation, resulting in an increased prevalence of RLS in PD [88]. Patients with PD may not report RLS symptoms unless asked because they regard RLS as a part of the PD symptom complex [89]. 

In PD patients, nonmotor symptoms related to nondopaminergic systems, such as cognitive impairments, autonomic dysfunction, depression, and sleepiness, but not motor symptoms were observed to be associated with RLS [90]. Gómez-Esteban et al. [85] reported a high prevalence of RLS (21.9%) in PD patients but determined no difference in disease severity, UPDRS scores, or quality of life between PD groups with or without RLS. Caution must be taken for RLS mimics in PD. Peralta et al. [91] described a positive association between motor fluctuations, the wearing-off phenomenon, and RLS symptoms in PD patients but suggested that off-period restlessness can be an “RLS mimic.” 

Autopsy studies have revealed increased substantia nigra (SN) iron levels in PD patients [92] and decreased SN iron levels in RLS patients [93]. A recent study by Kwon et al. [94] employing transcranial sonography demonstrated no significant differences in SN echogenicity, which is considered to reflect the quantity of tissue iron, between PD patients with and without RLS, whereas the idiopathic RLS patients demonstrated significant SN hypoechogenicity. This finding suggests that the pathogenesis of PD with RLS and idiopathic RLS may involve different mechanisms. 

Caffeine, alcohol, and several medications, including antihistamines, dopamine antagonists, tricyclic antidepressants, and serotoninergic reuptake inhibitors, can exacerbate RLS [95]. Although the pathophysiology of RLS is not fully understood, a central dopaminergic dysfunction has been implicated based on the findings that dopamine agonists relieve patients' symptoms and that decreased dopamine D2 receptor binding is observed in the striata of RLS patients by SPECT [96]. The A11 hypodopaminergic theory, which involves spinal cord positive feedback mechanisms that mediate dopamine, has been proposed using an animal model [97]. Dysfunctions of the A11 dopaminergic diencephalospinal pathways, which innervate the preganglionic sympathetic neurons and dorsal horn of the spinal cord, lead to an increased sympathetic drive that results in the occurrence of RLS [98]. An iron deficiency can also contribute to impairments in dopamine signaling in the brain. Low iron and ferritin levels in the cerebrospinal fluid have been observed in patients with RLS [99, 100]. Iron replacement therapy should be considered when serum levels of ferritin are below 50 *μ*g/L. When taken at bedtime, dopamine agonists such as pramipexole and ropinirole are an effective treatment for RLS. 

RLS and PD may share a pathogenesis; however, the pathogenic link between RLS and PD should be investigated further.

### 5.3. Sleep Apnea Syndrome

Previous studies have reported a high incidence of sleep apnea syndrome (SAS) in PD patients (approximately 20%–60%) compared with age- and sex-matched control patients [21, 101, 102]. In these studies, the body mass index of patients with PD was similar to or even lower than that of control patients, suggesting that upper airway muscle dysfunction caused by nocturnal akinesia or dyskinesia of the respiratory muscle may play a role in the development of obstructive sleep apnea (OSA) in PD [103]. By contrast, a study measuring PSG over three consecutive nights found that the apnea-hypopnea index (AHI) was not different between PD patients and control patients and that the rate of OSA in PD patients was similar to that observed in the general population [104]. Another study reported that sleep apnea, defined as an AHI >5, was less frequent in the PD group compared with an in-hospital control group who exhibited daytime sleepiness (27% versus 40%) and that daytime sleepiness was caused by other, nonapneic, mechanisms [105]. The relationship between OSA and PD requires further investigation. 

Nocturnal stridor, a life-threatening event caused by vocal cord abductor dysfunction, has been observed in patients with PD but occurs more frequently in patients with multiple system atrophy [106]. It is important to screen for vocal cord abductor dysfunction using laryngoscopy during sleep. Adequate treatments, including continuous positive airway pressure therapy, noninvasive positive pressure ventilation, or tracheotomy, can prevent sudden death in patients.

### 5.4. Circadian Rhythm Sleep Disorders

In PD, circadian rhythm during sleep, blood pressure, heart rate, and levels of cortisol and melatonin hormones may be altered, possibly due to autonomic dysfunction, changes in sleep structure, and dopaminergic treatments [107–110]. In addition, PD patients with dementia may exhibit sundowning [111]. The suprachiasmatic nucleus (SCN) is the crucial center responsible for generating the circadian rhythm. The SCN is included in the paraventricular zone of the hypothalamus, which is a component of the central autonomic network (CAN) that controls autonomic functions in a state-dependent manner [112]. The SCN seems to be intact in PD patients, but the reported involvement of the hypothalamus and brainstem in PD patients [113, 114] appears to be associated with the CAN. 

In animal models of PD, significant decreases in midline estimating statistics of rhythm and phase advances were observed for heart rate, locomotor activity, and core body temperature (CBT) [115]. Pierangeli et al. [116] have demonstrated that a nocturnal fall in CBT was attenuated in multiple system atrophy compared with PD. Similarly, in a small cohort study of progressive supranuclear palsy patients, a decreased circadian amplitude of CBT was observed compared with that of PD patients [117]. In our previous study of 24 nondepressed PD patients and six depressed PD patients, we demonstrated that nondepressed patients exhibited a circadian rhythm of CBT, but two out of six of the depressed patients exhibited an infradian rhythm as a predominant rhythm in CBT. The remaining depressed patients demonstrated a decreased circadian amplitude of CBT compared with that of the nondepressed PD patients [118]. Our results suggest that PD patients with depression can exhibit circadian rhythm abnormalities compared with PD patients without depression. Further research, including a larger number of PD patients and control subjects, is needed to confirm this finding.

### 5.5. Depression

The reported prevalence of depression in PD patients varies, ranging from 2.7% to 89% [119]. This variation may be due to the population studied or methods employed for diagnosis. Depression is associated with sleep disorders, nocturnal motor symptoms, and poor quality of life [120–123]. Depression is also related to motor fluctuations, such as wearing-off symptoms [124]. In a recent randomized, double-blinded, placebo-controlled trial, pramipexole, which has a higher affinity for D3 than D2 or D4 receptors, significantly improved the depressive symptoms of PD patients, suggesting that this drug is useful for treating depression in addition to the selective serotonin reuptake inhibitors and tricyclic antidepressants often used for treating depression [125]. Hence, identifying and treating depression or depressive symptoms can be beneficial in ameliorating sleep disorders and nocturnal motor dysfunctions in PD patients.

## 6. Conclusion

Sleep disorders can occur in the early stages of PD and worsen as the disease progresses. The worsening of sleep disturbances occurs in a manner similar to the progression of motor dysfunctions, cognitive impairments, and depression, which supports the idea that complex mechanisms and impairments of the arousal system and sleep structure play a role. Sleep disturbances can be underestimated if the patients, their families, and their physicians do not investigate the possibility of impaired sleep. 

Active intervention for sleep disturbance is of great importance, as sleep disturbances can significantly impair the quality of life of patients with PD.
